# Chemokine (C–C motif) receptor 2 is associated with the pathological grade and inflammatory response in IgAN children

**DOI:** 10.1186/s12882-022-02839-y

**Published:** 2022-06-20

**Authors:** Yanjie Shen, Zhiqing Zhu, Rui Wang, Lili Yan, Shuaichen Sun, Ling Lu, Zhenhua Ren, Qin Zhang

**Affiliations:** 1 Department of Anatomy, Anhui Medical University, 81 Meishan Road, Hefei, 230032 Anhui China; 2 Department of Pediatrics, First Affiliated Hospital of Anhui Medical University, 19Th Floor of Medicine and Medical Tech Building, 218 Jixi Road, Hefei, 230022 Anhui China

**Keywords:** Chemokine (C–C motif) receptor 2, Inflammatory factors, Mesangial cells, IgAN, Children

## Abstract

**Background:**

Chemokine (C–C motif) receptor 2 (CCR2) is involved in important physiological and pathological processes, such as inflammation and autoimmune diseases. Abnormal immune and inflammatory responses play a critical role in the development and progression of IgA nephritis (IgAN). However, the role of CCR2 in IgAN is unknown.

**Methods:**

Fifteen IgAN children who were diagnosed by kidney biopsy provided kidney biopsy tissue, blood and urine samples, and age-matched healthy control subjects (blood donators *n* = 12; tissue donators *n* = 8) were included. Immunohistochemical analysis was used to detect the expression of CCR2, MCP-1, IL-6, IL-17, and TNF-α in the kidney tissues. Relative optical density (OD) was calculated by Image J software, and the correlation between CCR2 expression and pathological grade in IgAN children was analyzed.

**Results:**

The expression of CCR2 significantly increased in mesangial cells of children with IgAN compared to that in control group (*P* < 0.001), especially in IgAN patients with Lee’s grade III to IV (*P* < 0.001). Interestingly, CCR2 expression was positively correlated with Lee’s grade (*r* = 0.9152, *P* = 0.0001) in IgAN children. The expression levels of inflammatory factors were markedly increased in IgAN children, and importantly CCR2 expression was positively correlated with it’s expression level.

**Conclusions:**

The results suggest that CCR2 signaling might be involved in pathological process and inflammatory responses of children IgAN, and could potentially be an intervention target in children IgAN.

**Supplementary Information:**

The online version contains supplementary material available at 10.1186/s12882-022-02839-y.

## Introduction

IgA nephropathy (IgAN) was first discovered and described by Berger and Hinglais in 1968 [1]. Clinical manifestations are microscopic hematuria, or gross hematuria, and proteinuria. Kidney pathology shows the deposition of IgA immune complexes, leading to glomerulonephritis characterized by mesangial cells and stromal hyperplasia. IgAN is one of the most common types of primary glomerulonephritis in China, accounting for about 45% of primary glomerulonephritis. IgAN can occur at any age and is common in children and adolescents [2, 3]. About 5–40 years after diagnosis, about 20% -40% progress to kidney failure [4].

The pathological classification of IgAN includes Lee's classification, Hass classification, and Oxford classification, which are important guidelines for the treatment and prediction of IgAN [5]. Lee’s classification considers histologic lesions that may predict the prognosis of IgAN, and is easy to operate and apply [6]. Oxford Classification Working Group through univariate analysis proved that mesangial proliferation, segmental glomerulosclerosis, and tubular atrophy/interstitial fibrosis are independent high-risk factors for the progression of IgAN [7]. So far, the pathogenesis of IgAN is still unclear, but the immune pathogenesis of IgAN has been widely studied and reported [8, 9]. Galactose deficiency-IgA1 (Gd-IgA1) complex deposits in the mesangium region [10], and the mesangial cells proliferation are the common pathological changes in IgAN [11]. In addition, damaged mesangial cells release pro-inflammatory factors, such as IL-6, MCP-1, TNF-α and so on, which promotes the inflammatory response, and further aggravates kidney damage [12].

Chemokine (C–C motif) receptor 2 (CCR2) is a G protein-coupled receptor, a protein of 355 amino acid residues, which is mainly expressed in leukocytes, endothelial cells, macrophages, and smooth muscle cells, etc. [13]. Chemokines and their receptors are involved in important physiological and pathological processes, such as inflammation and autoimmune diseases, transplant rejection, tumor growth and metastasis [14, 15]. Previous report has shown an association between monocyte chemoattractant protein-1 (MCP-1) and CCR2 gene polymorphisms and higher grade histopathology of IgAN by Lee's classification [16]. Animal experiment results show that CCR2 autoimmune-deficient mice have significantly reduced lupus nephritis [17]. In addition, many previous studies showed that the blockade of chemokine (C–C motif) ligand 2 (MCP-1) and its receptor CCR2 signaling pathway reduced inflammatory cytokines, such as IL-6 and TNF-α [18, 19]. However, so far the role of CCR2 in the pathology and inflammation of IgAN is unknown. This study investigated the expression of CCR2 in kidney tissues of children with IgAN, and its correlation with Lee's grade and the expression levels of inflammatory factors in kidney tissues.

## Materials and methods

### Reagents

Rabbit anti-CCR2 antibody was obtained from BioVision (Milpitas, CA). Rabbit anti-MCP-1, rabbit anti-IL-17, mouse anti-IL-6 and rabbit anti-TNF-α antibodies and were obtained from AbCam (Cambridge, MA). Pre-immune rabbit serum, secondary antibodies (PV-9000 kit) and DAB Detection Kit were purchased from Zsbio (Beijing, CHN). FITC-labeled anti-human IgA was purchased from Zsbio (Beijing, CHN). Other chemicals and reagents were purchased from Zsbio (Beijing, CHN).

### Clinical specimen collection

Fifteen children with a diagnosis of IgAN presenting to the Department of Pediatrics, First Affiliated Hospital of Anhui Medical University, were enrolled between July 2014 to September 2017. All children were diagnosed with primary IgAN by kidney biopsy [20]. The procedure for the kidney biopsy was performed as previously described with some modifications [21], and the pathological staining was shown in the supplementary materials (sFigure 1 and sFigure 2). All patients did not receive the treatment with steroid or other immunosuppressant agents before kidney biopsy. All children were excluded from secondary IgAN caused by allergic purpura nephritis, lupus nephritis, or hepatitis B virus-related nephritis. Eight control kidney tissue specimens were taken from children with kidney surgery because of other kidney diseases, such as kidney duplication. Wax block specimens of kidney biopsy were used for subsequent immunohistochemical staining. Twelve control blood and urine specimens were taken from normal children with health checkup. Study protocol was approved by the hospital institutional review board of the First Affiliated Hospital of Anhui Medical University, and the number of the ethics approval was NO. 20140235. Kidney biopsy specimens from patients and control group had obtained the informed consent of the patient's family.

### Immunohistochemistry

The wax block of kidney biopsy tissue and control kidney tissues were cut at 5 μm thickness sections. The procedure for immunohistochemistry has been previously described [22]. Briefly, the sections were treated with conventional dewaxing tissue sections to water, and sodium citrate buffer solution antigen repair, and 3% H_2_O_2_ to reduce the non-specific staining, and 5% BSA was added to block endogenous antigen. The slides were incubated with rabbit anti-CCR2 (dilution 1:200), or free-immune serum (1:50), rabbit anti-MCP1 (1:100), IL-17 (1:100), TNF-ɑ (1:100) and mouse anti-IL-6 (1:500) overnight at 4 °C. After rinsing in PBS, the sections were incubated with peroxidase-conjugated goat anti-rabbit or anti-mouse IgG at room temperature for 1 h. After PBS washing for three times, DAB color solution dropped onto the section, and incubated for 3–5 min. Then the section was stained with hematoxylin for 2 min. After drying and transparent, the section was observed under microscope. The analysis of specific staining was performed using Image J software (NIH, Bethesda, MD, USA). Negative controls were performed by replacing the primary antibody with free-immune serum, and the results was shown in the supplementary materials (sFigure 3). The relative optical density (OD) was counted at 200 × magnification, and the positive signaling were counted in twenty randomly selected sections using the software of Image J v1.8.0 (National Institutes of Health, Bethesda, MD).

### Blood and urine testing

The serum and urine samples were obtained by centrifugation, and 20ul was used to measure urine red blood cell count (URBC), 24-h urine protein (UPro), blood urea nitrogen (BUN), and serum creatinine (SCr) were measured on Abbot architect ci8200 analyser (Abbott, Abbot Park, IL, USA). Each sample was tested 3 times, and the average value was used for statistical analysis.

### Statistical analysis

Graph Pad Prism 5 software was used to plot and process the experimental data. All data were presented as the means ± SEM. Differences between two groups were analyzed by Student's t test for unpaired data. ANOVA followed by Scheffé’s post-doc test was used in multi-group analysis. We performed Spearman’s correlation tests to specify relationships among the variables. *P* values of < 0.05 were considered significant.

## Results

### Clinical features of children with IgAN

Fifteen IgAN children, including 10 males and 5 females (mean age 11.67 ± 1.76 years old, absolute range 7.4–13.2 years), were enrolled in the study. Eight control kidney tissue specimens were taken from children, including 5 males and 3 females (mean age 10.75 ± 2.24 years old, absolute range 6.3–14.5 years), with normal pathological examination after kidney disease. Twelve control blood and urine specimens were taken from normal children with health checkup.

All children with IgAN had gross hematuria or microscopic hematuria, and the urinary red blood cell count was 689 ± 427 cells per μl, which was significantly higher than control group (*P* < 0.05). Twelve control blood and urine specimens taken from normal children, including 8 males and 7 females (mean age 11.64 ± 3.17 years old, absolute range 6.8–15.6 years), were detected for UPro, URBC, SCr and BUN. Compared with the control group, UPro and URBC of the children were significantly increased in IgAN children (*P* < 0.01), and no significant difference were found in serum BUN and SCr (*P* > 0.05), as shown in sTable 1. Blood test and liver function tests were normal in IgAN children (*P* > 0.05), and the data were not shown.

### CCR2 expression increased significantly in IgAN children

**Fig. 1:**
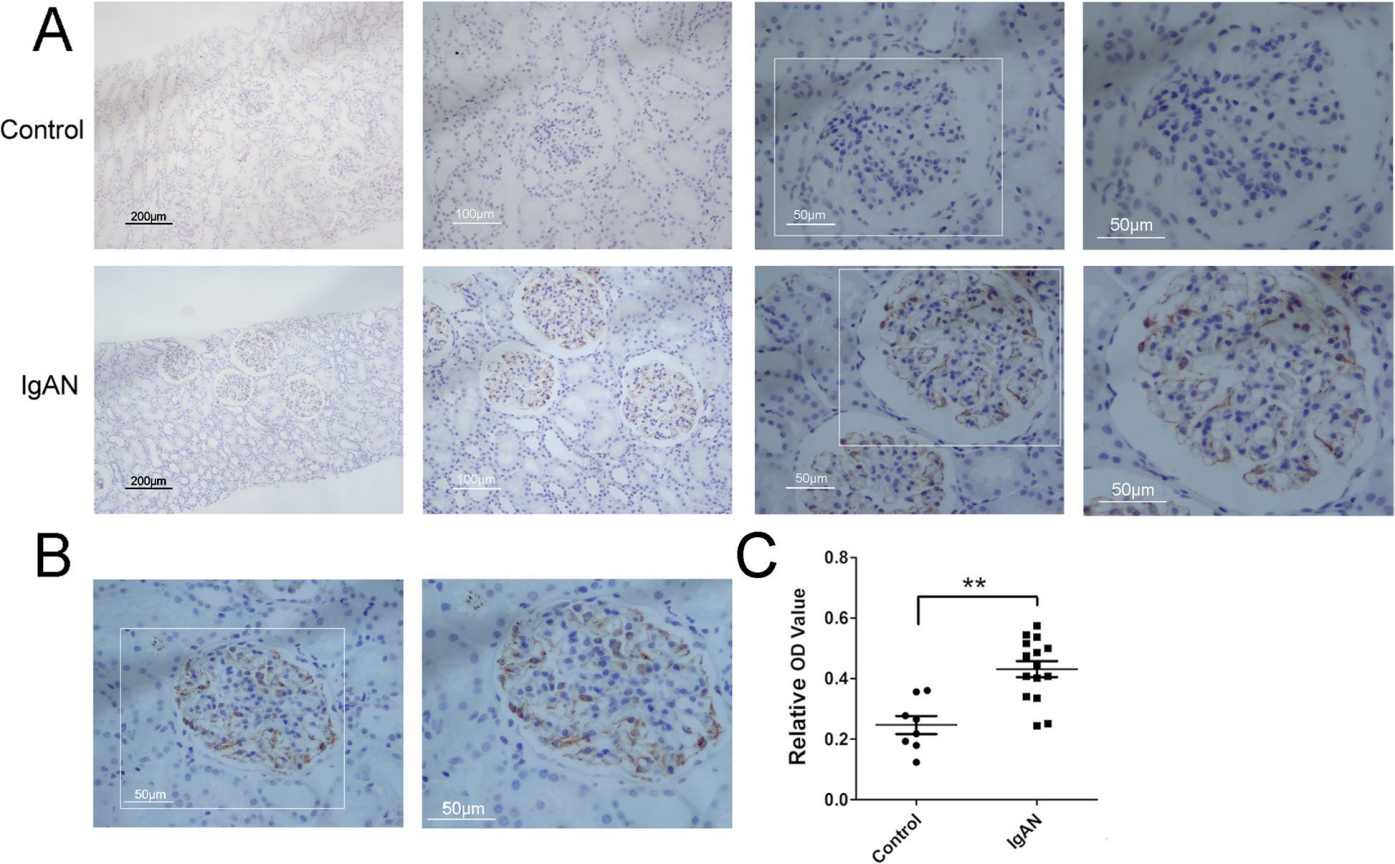
CCR2 expression in glomerular mesangial cells in IgAN children. Expression of CCR2 in kidney tissue of controls (**A**: upper row) and IgAN children (**A**: lower row) was detected by DAB staining. In the last column of **A** and **B** showed single glomerular cross sections. The Bar of first column from left in **A** was 200 µm, and then second column was 100 µm, and the third, last column and **B** was 50 µm. Relative optical density (OD) of kidney CCR2 expression in controls and IgAN patients (**C**) was calculated by Image J software. Compared with control group, **P < 0.01

**Fig. 2:**
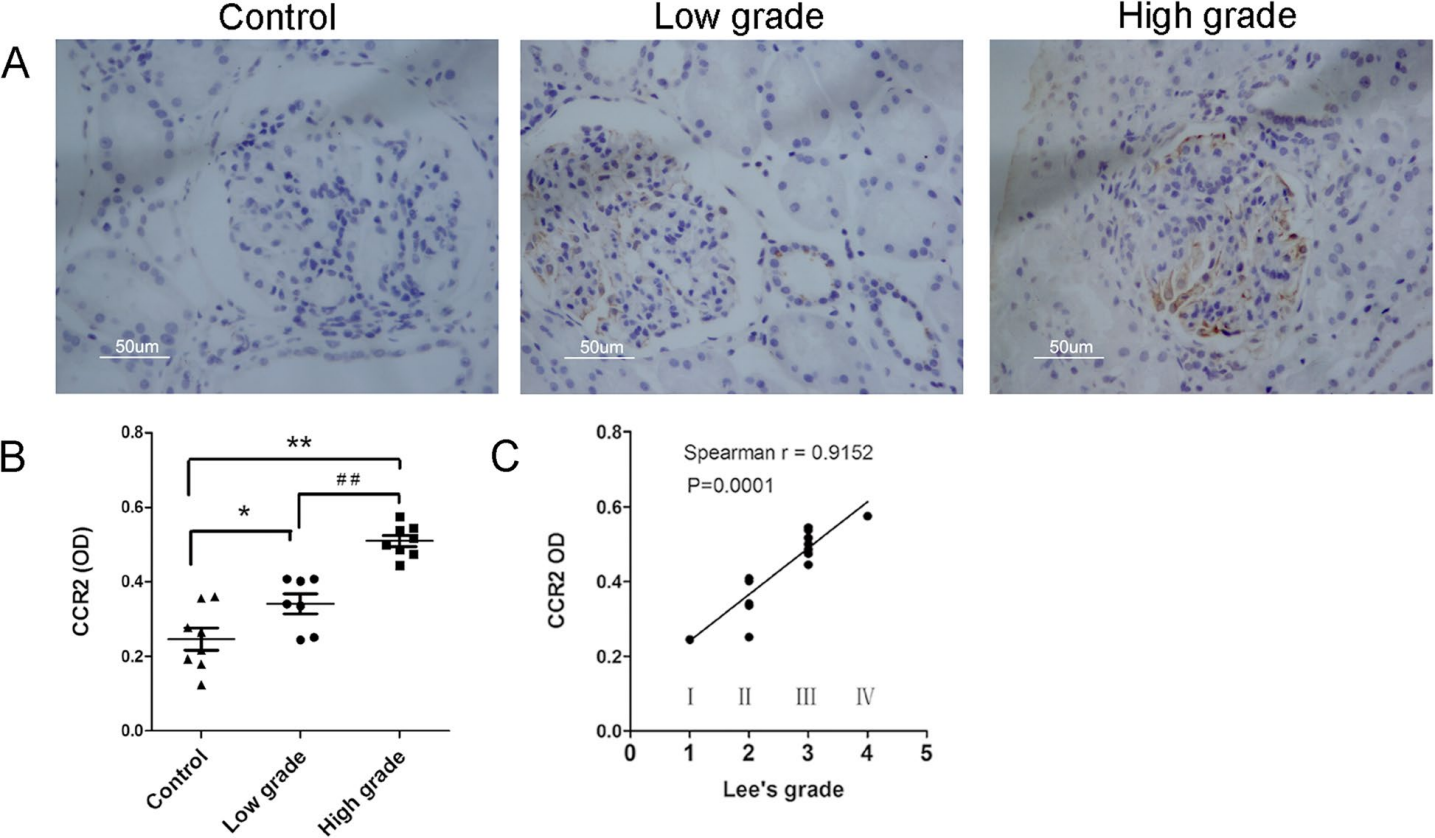
The correlation between CCR2 and Lee’s grade in IgAN children. Fifteen patients were divided into 2 groups, low grade group (Lee’s grade I to II) (*n* = 7, **A**: middle), and high grade group (grade III to IV) (*n* = 8, **A**: right). The Bar of **A** was 50 µm. Image J analysis software was used to calculate the relative optical density (OD) of CCR2 DAB staining in control group, low and high grade group (**B**). The correlation analysis between the relative OD value of CCR2 and Lee’s grade was performed by Spearman’s correlation tests (**C**). Compared with control group, *P < 0.05, **P < 0.01; Comparison between Lee’s grade groups,.^# #^*P* < 0.01

As shown in the Figs. 1A and 2A, CCR2 was rarely observed in the kidney tissues of normal control group. However, CCR2 expression increased significantly in IgAN children (Figs. 1A and 2A). CCR2 positive signals were concentrated in the glomeruli, while in the renal tubular were rare (Figs. 1A, B and 2A). Most of the CCR2 positive signals were expressed in the glomerular mesangial cells of IgAN children, whereas in the vascular endothelial cells were rare (Figs. 1A and B). As shown in Fig. 1C, compared with normal control group, the relative optical density (OD) of CCR2 positive signals calculated by the software (Image J) had significantly increased in IgAN children (*P* < 0.001).

### CCR2 expression was positively correlated with Lee’s grade in IgAN children

According to Lee's classification of IgAN [6, 20], the renal pathology of 15 children was classified, including 1 case of grade I, 6 cases of grade II, 7 cases of grade III, and 1 case of grade IV. We had divided 15 patients into 2 groups, low grade group (Lee’s grade I to II) (*n* = 7), and high grade group (Lee’s grade III to IV) (*n* = 8). In IgAN children of high grade group, CCR2 positive signals were significantly higher than that of low grade group (Fig. 2A). Quantitative analysis of optical density (OD) also confirmed the above results (*P* < 0.001) (Fig. 2B). Interestingly, we found that the relative OD value of CCR2 had a significant positive correlation with the pathological grade of Lee’s classification in IgAN children (*r* = 0.9152, *P* = 0.0001) (Fig. 2C). However, in 15 cases of children, we did not find any correlations between Lee’s grade and IgA deposition (*r* = 0.2322, *P* = 0.4050), as shown in sFigure 2B. Furthermore, there was no correlation between IgA deposition and the relative optical density (OD) of CCR2 expression analyzed by DAB staining in IgAN children (*r* = 0.2783, *P* = 0.3152) (sFigure 2C).

### CCR2 expression was positively correlated with the expression levels of inflammatory factors

**Fig. 3:**
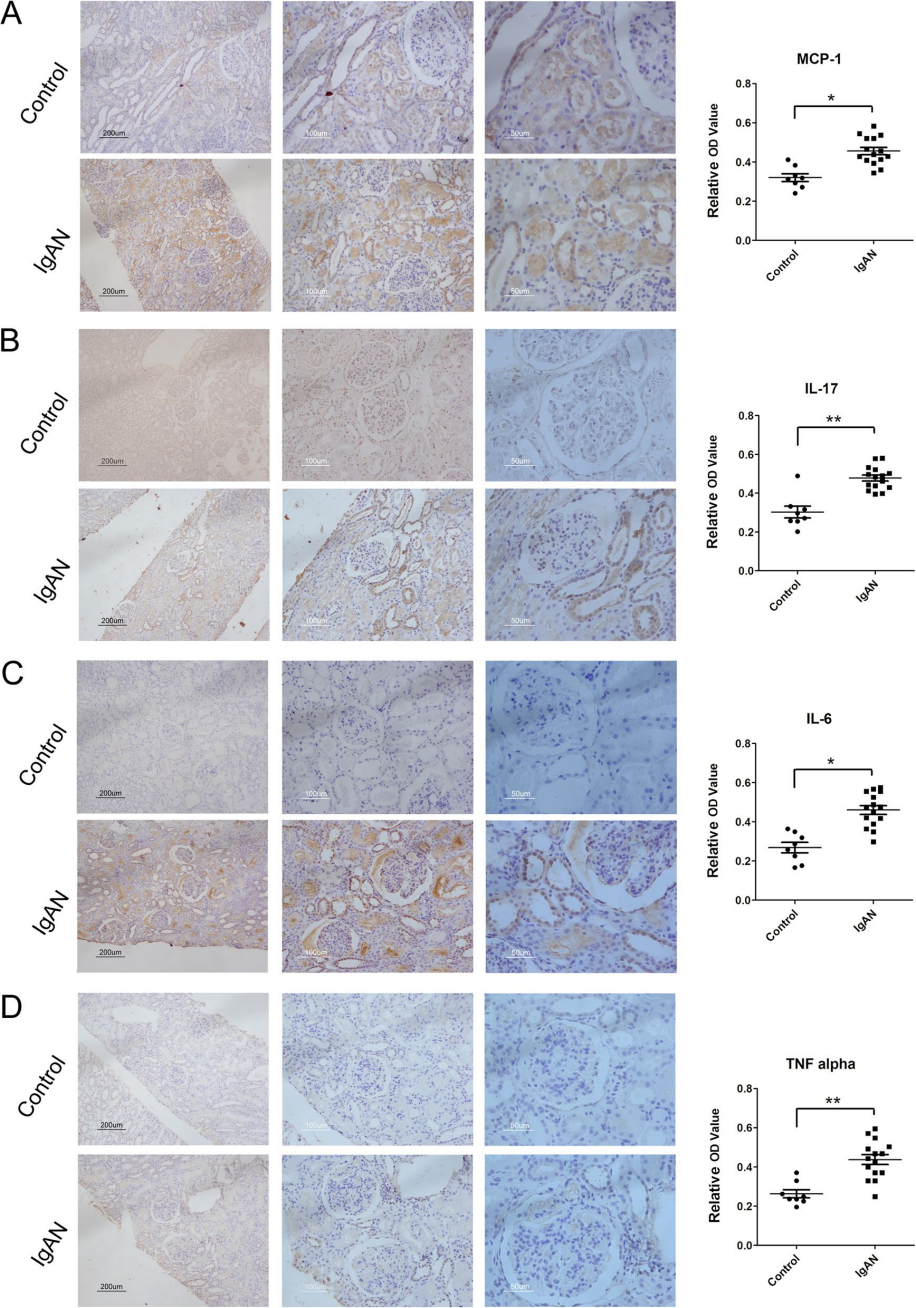
The expression levels of inflammatory factors in IgAN children. Expression of MCP-1 (**A**), IL-17 (**B**), IL-6 (**C**) and TNF-α (**D**) in kidney tissue of control and IgAN children was detected by DAB staining. The Bar in left column of **A-D** was 200 µm, and in the middle and right column respectively was 100 µm and 50 µm. The relative OD value of MCP-1 (**A**), IL-17 (**B**), IL-6 (**C**) and TNF-α (**D**) were measured by Image J analysis software. Compared with control group, *P < 0.05, **P < 0.01

Moreover, the expression levels of inflammatory factors were detected by immunohistochemistry, including MCP-1, IL-17, IL-6 and TNF-α (Fig. 3). As shown in Fig. 3A, MCP-1 expression was mainly concentrated in the renal tubules, especially in the epithelial cells of collecting duct, while IL-17 (Fig. 3B), IL-6 (Fig. 3C) and TNF-α (Fig. 3D) were mainly concentrated in the glomerulus and collecting duct. Compared with the normal control group, MCP-1, IL-17, IL-6 and TNF-α increased significantly in the kidney tissue of IgAN children (*P* < 0.05) (Fig. 3A-D), and the similar results were found in the relative optical density (OD) of MCP-1, IL-17, IL-6 and TNF-α (Fig. 3A-D).

**Fig. 4:**
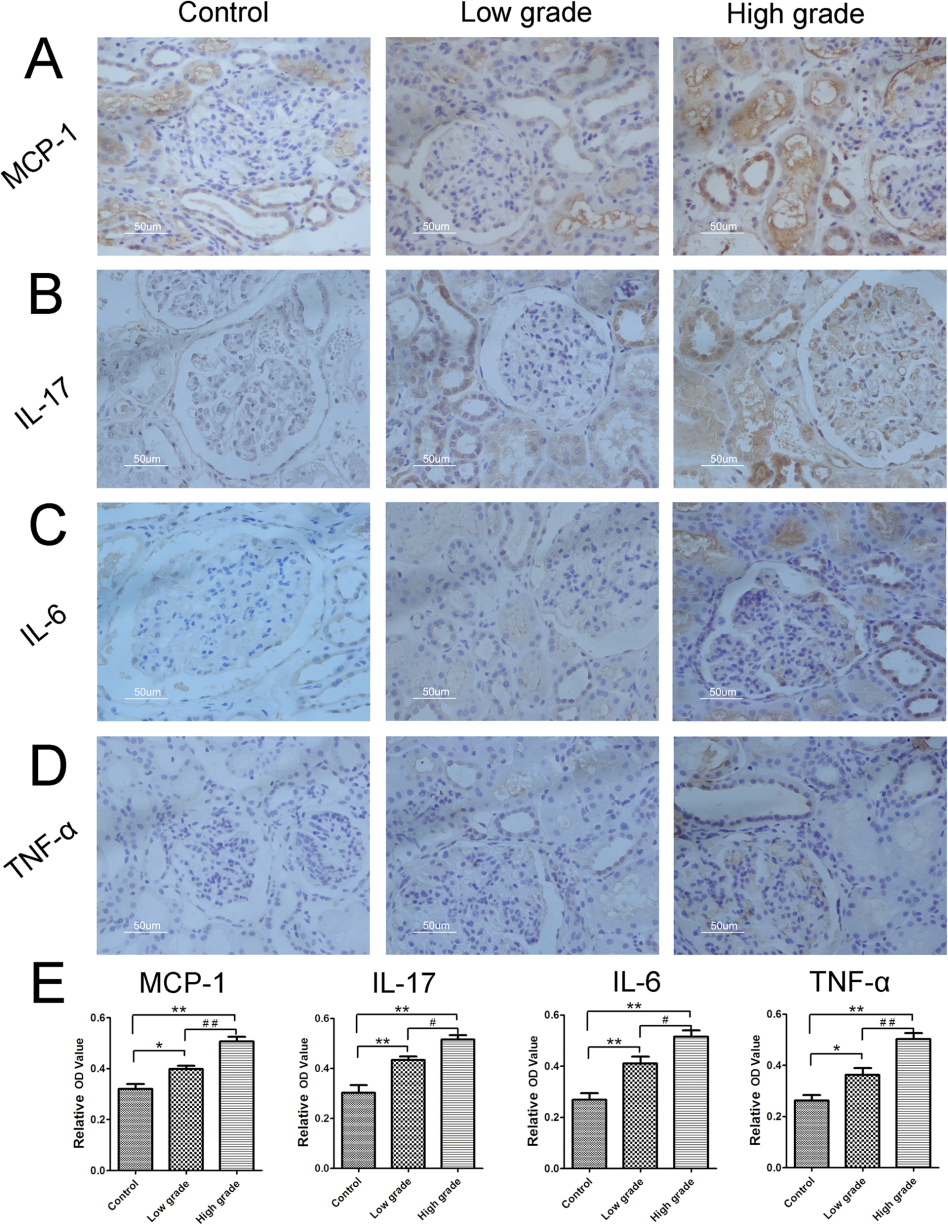
The expression levels of inflammatory factors in different Lee’s grade of IgAN children. Expression of MCP-1 (**A**), IL-17 (**B**), IL-6 (**C**) and TNF-α (**D**) in kidney tissue of control, and low grade group (Lee’s grade I to II) (*n* = 7, middle column) and high grade group (grade III to IV) (*n* = 8, right column) was detected by DAB staining. The Bar in **A-D** was 50 µm. Image J analysis software was used to calculate the relative optical density (OD) in control group, low and high grade group (**E**). Compared with control group, *P < 0.05, **P < 0.01; Comparison between Lee’s grade groups, ^#^*P* < 0.05, ^# #^*P* < 0.01

**Fig. 5:**
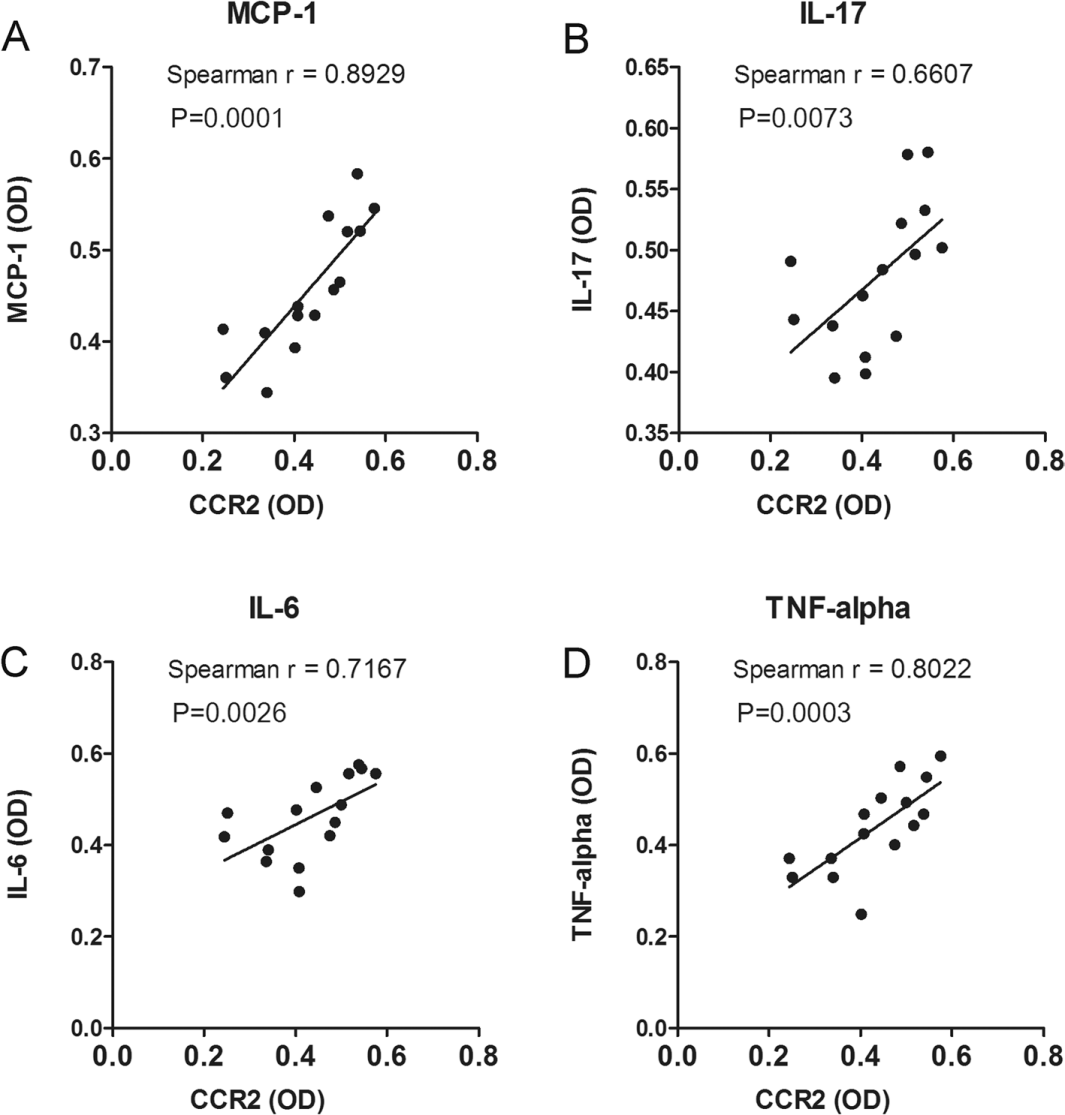
The correlation between CCR2 and inflammatory factors in IgAN children. The correlation analysis between the relative OD value of CCR2 and MCP-1 (**A**), IL-17 (**B**), IL-6 (**C**) or TNF-α (**D**) were analyzed by Spearman’s correlation tests

As shown in Fig. 4, the expression levels of inflammatory factors in high grade group (Lee’s grade III to IV) were significantly higher than that of low grade group (Lee’s grade I to II) (*P* < 0.05). Furtherly, the relationships between CCR2 expression and the expression levels of inflammatory factors were also studied. We found that CCR2 expression had a significant positive correlation with the expression levels of MCP-1 (*r* = 0.8929, *P* = 0.0001), IL-17 (*r* = 0.6607, *P* = 0.0073), IL-6 (*r* = 0.7167, *P* = 0.0026) and TNF-α (*r* = 0.8022, *P* = 0.0003) in IgAN children (Fig. 5).

## Discussion

IgA nephropathy (IgAN) is the most common form of primary glomerulonephritis worldwide, which may occur at any age, and is most common in children and adolescents [2, 3]. IgAN is a frequent cause of kidney failure, and 20–40% of IgAN patients eventually progress to kidney failure within 20 years of onset [23, 24]. At present, the immune pathogenesis of IgAN has been widely studied and reported, but the specific pathogenesis is still unclear [8, 9].

The role of chemokines in the pathogenesis of IgAN has gradually attracted attention [17, 25]. Chemokine (C–C motif) receptor 2 (CCR2) is a G protein-coupled receptor with a 355 amino acid residue protein, which is mainly expressed in leukocytes, endothelial cells, macrophages and smooth muscle cells [26]. CCR2 is the receptor of MCP-1 (monocyte chemoattractic protein 1), and play an important role in inflammatory disorders [16, 27]. MCP-1/CCR2 signaling was involved in human crescentic glomerulonephrtitis and murine lupus nephritis [28, 29]. Previous reports have shown that MCP-1 and CCR2 gene polymorphisms may affect the progression of IgAN [16]. Previous study has shown that CCR2 activation plays an important role in the development of hypertensive nephropathy via increased oxidative stress and inflammation [30]. Several reports have shown that glomerulosclerosis and tubulointerstitial fibrosis were significantly ameliorated in CCR2-/- mice with adriamycin nephropathy, and accompanying with the reduction of macrophage and fibrocyte infiltration and inflammation in glomeruli and the tubulointerstitium [31–33]. These evidences indicate that CCR2 mediate inflammation, and cause collagen accumulation, and promote renal fibrosis, and ultimately cause severe kidney damage [32]. However, the role of CCR2 in IgAN is not yet clear.

In this study, we found that CCR2 positive signals were only expressed in the glomeruli of children with IgAN, and importantly most of the CCR2 positive signals were expressed in glomerular mesangial cells. Interestingly, we found that CCR2 expression was positively correlated with Lee’s grade in IgAN children, and CCR2 positive signal in children with Lee’s pathological grade III to IV was significantly higher than that of Lee’s grade I to II (*P* < 0.01). Although our study only included 15 children with IgAN, the exciting thing is that we found that CCR2 expression had a significant positive correlation with Lee’s grade (*P* = 0.0001) (Fig. 2C). We hypothesize that Gd-IgA deposition stimulates mesangial cells, and up-regulates CCR2 expression. Elevated CCR2 could increase the expression of multiple cytokines and chemokines, such as MCP-1, IL-6 and TNF alpha, and induces mesangial cell proliferation and matrix deposition, and further aggravate kidney damage [16, 34]. Therefore, CCR2 may play an important regulatory role in the pathological process and inflammatory response of children IgAN.

It has been reported that histological features of IgAN on diagnostic biopsy are different in children and adults [35]. Children IgAN are more likely to have mild histologic lesions and less advanced chronic lesions, such as glomerulosclerosis and interstitialfibrosis, compared to adults [36–38]. Therefore, previous physicians have long believed that children IgAN is a benign disease to remission, and occasionally to late relapse during adulthood [39, 40]. However, recent statistics show that the prognosis of children with IgAN is as serious as that of adults [39, 41, 42]. Recent studies show that children IgAN presents with more acute mesangial proliferation and inflammation, while in adults it correlates with chronic lesions and nephron reduction [39]. Proteinuria in children with IgAN appears to be due to acute mesangial proliferation and inflammation [35].

In our study, 24 h Upro is significantly increased in children IgAN (*P* < 0.01), and the expression of chemokines and cytoinflammatory factors in the kidney tissue of children with IgAN increased significantly, such as MCP-1, TNF alpha, IL-6 and IL-17, which are similar to previous studies [12, 43]. Interestingly, we found that the expression levels of inflammatory factors in children with Lee’s pathological grade III to IV was significantly higher than that of Lee’s grade I to II (*P* < 0.01). These results indicate that chemokines and cytoinflammatory factors play an important role in the pathological changes of IgAN.

Chemokines and cytoinflammatory factors are assumed to play an important role as mediators of inflammation and as progression factors in various kidney disorders [44–46]. Various cytokines, such as IL-6 and TNF-α, have been increased in the kidney tissues of patients with IgAN, which contributes to further glomerular injury [46, 47]. Previous study has shown that MCP-1 can activate the NF-κB pathway by binding to CCR2 to promote the secretion of inflammatory factor IL-6 [48]. Similarly, MCP-1 can also stimulate vascular endothelial cells to produce IL-6 through this pathway, and promote the development of glomerulonephritis [49]. Interleukin 17 (IL-17) plays an important role in the pathogenesis of autoimmune diseases [50]. Recently, the role of Th17 cells producing proinflammatory interleukin 17A (IL-17A) was established in the pathogenesis of various glomerulonephritis [51]. Therefore, IL-17A is considered as a potential biomarker of IgA nephropathy [52, 53]. In our study, MCP-1, TNF alpha, IL-6 and IL-17 in the kidney tissue of children with IgAN increased significantly. Importantly, we found that CCR2 signal was positively correlated with the expression levels of inflammatory factors. The results indicate that CCR2 signal may play an important role in the inflammatory response of IgAN.

In summary, we report that CCR2 expression is significantly increased in glomerular mesangial cells of IgAN children, especially in IgAN patients with Lee’s grade III to IV. Importantly, CCR2 expression is positively correlated with Lee’s grade and the expression levels of inflammatory factors in IgAN children. These results suggesting that CCR2 signal is associated with the pathological grade and the inflammatory response in children IgA nephropathy, indicating that CCR2 plays a pathogenic role in IgAN, and targeting CCR2-associated signaling pathways may be a strategy for the treatment of IgAN. However, further mechanistic studies are needed to fully explore the roles of CCR2 in IgAN.

## Supplementary Information

**Additional file 1: sTable 1. **Demographics and clinical characteristics of patients in IgAN group and controls. **sTable 2. **Lee’s grade and Oxford Classification scores characteristics of patients in the IgAN children. **sTable 3.** The relationship of Lee’s grade and Oxford classification. **sTable 4.** The relationship of Lee’s grade and Oxford classification. **sFigure 1.** Kidney pathological staining in IgAN children. **sFigure 2. **IgA immunofluorescence staining in IgAN children. **sFigure 3.** Control immunohistochemistry staining. **sFigure 4.** The relationship between CCR2 expression and clinical presentation. **sFigure 5.** The correlation between Oxford classification and inflammatory factors. **sFigure 6.** The correlation between mesangial hyperplasia and inflammatory factors. **sFigure 7.** The correlation between endothelial cell hyperplasia and inflammatory factors.

## Data Availability

All data generated or analyzed during this study are included in this published article and its supplementary information files.

## Abbreviations

BUN: Blood urea nitrogen
CCR2: Chemokine (C–C motif) receptor 2
Gd-IgA1: Galactose-deficient IgA1
IgA: Immunoglobulin A
IgAN: IgA nephropathy
IL-6: Interleukin-6
IL-17: Interleukin-17
MCs: Mesangial cells
MCP-1: Monocyte chemoattractant protein-1
OD: Optical density
Cr: Creatinine
TNF-α: Tumor necrosis factor alpha
UPro: 24-Hour urine protein
