# Supplementary material for: Chemokine (C–C motif) receptor 2 is associated with the pathological grade and inflammatory response in IgAN children
Source: BMC Nephrol. 2022 Jun 20;23:215. doi: 10.1186/s12882-022-02839-y (PMC9210650; doi:10.1186/s12882-022-02839-y)
Supplement: Supplementary file 1 — Additional file 1: sTable 1. Demographics and clinical characteristics of patients in IgAN group and controls. sTable 2. Lee’s grade and Oxford Classification scores characteristics of patients in the IgAN children. sTable 3. The relationship of Lee’s grade and Oxford classification. sTable 4. The relationship of Lee’s grade and Oxford classification. sFigure 1. Kidney pathological staining in IgAN children. sFigure 2. IgA immunofluorescence staining in IgAN children. sFigure 3. Control immunohistochemistry staining. sFigure 4. The relationship between CCR2 expression and clinical presentation. sFigure 5. The correlation between Oxford classification and inflammatory factors. sFigure 6. The correlation between mesangial hyperplasia and inflammatory factors. sFigure 7. The correlation between endothelial cell hyperplasia and inflammatory factors. [file 12882_2022_2839_MOESM1_ESM.docx]

**Supplementary materials**

**1. Methods**

1.1 HE staining

Paraffin sections were dewaxed to water, and then the slices were stained with hematoxylin for 5min. After rinsing under the tap, the slices were put into 1% hydrochloric acid alcohol for 2 seconds, and then the sections were rinsed (using running water) for 30 min. Next, the sections were incubated with eosin for 3 min. After washing several times, the sections were dehydrated with gradient alcohol, and transparented with xylene overnight, and sealed with neutral gum. The sections were examined under microscope, and the images were collected and analyzed, and quantified using Image J software (NIH, Bethesda, MD, USA).

1.2 PAS staining

The treatment process for paraffin sections was the same as HE staining. The sections were incubated with 10g/L iodic acid for 15-20min, and then rinsed with distilled water, and left to dry. Next, the sections were incubated with placesheff dye in room temperature for 30-60min. After wishing under running water for 5min, the slices were stained with hematoxylin for 3min. After wishing several times, dehydration, mounting and examination under microscope were the same as HE staining.

1.3 PASM staining

The treatment process for paraffin sections was the same as the above. The slices were put into potassium dichromate overnight. After slightly washing with water, the sectios were incubated with 1% iodic acid oxidation for 10-15 min at 72 °C. After wishing, the sections were stained with hexamine silver dye for 30-50 min. 2% gold chloride was added to the sections, and then the sections were washed with 5% sodium thiosulfate. Next, the sections were put into hematoxylin for 1-2 min, and immersed in 1% hydrochloric acid alcohol for differentiation and 1% aqueous ammonia. Then, the sections were stained with Masson dye solution for about 10min. After dehydration and mounting, collagen fibers appeared as dark strands under microscope.

1.4 Masson staining

After dewaxing and wishing with water, the sections were immersed in 0.5% cymbidium sinensis for 5-6 minutes. Then, the sections were stained with hematoxylin for 5 minutes. After washing with water, the sections were incubated with 1% richlet red: 2% acid magenta (1:2) for 15 min. Next, the sections pass through phosphomolybdate and 0.5% glacial acetic acid for several seconds. After staining with 1% light green for 3 minutes, the sections were through phosphomolybdate and 0.5% glacial acetic acid for several seconds. Images were collected under a microscope.

1.5 Control immunohistochemistry staining

According to the immunohistochemical staining procedure, the sections were treated with conventional dewaxing tissue sections to water, and followed by conventional antigen retrieval, endogenous antigen blocking. As the negative control, pre-immune rabbit serum (1:50) replaced primary antibody, and incubated with the section of kidney biopsy tissue form IgAN children patient overnight at 4°C. In addition, the slides of rat kidney tissue were incubated with rabbit anti-CCR2 (dilution 1:200) as the positive control. The following immunohistochemistry procedure is the same as previously described [[1](#_ENREF_1)].

1.6 Immunofluorescent staining

The procedure of immunofluorescent staining has been previously reported [[2](#_ENREF_2)]. Briefly, wax block of kidney tissue were sectioned at 5μm, and conventional dewaxing tissue sections to water. Cultured cells were fixed for 15 min in 4% paraformaldehyde. Then, the section and cells were permeabilized with 0.5% Triton X-100 in PBS for 30 minutes, and blocked with 5% BSA for 1h at room temperature, and incubated with primary antibodies overnight at 4˚C. After three times washing in PBS, the slides and cells were incubated with fluorescent secondary antibodies in the dark at room temperature for 1 h. Then, the slides and cells were washed three times in PBS, and added mounting media with DAPI. Pictures were taken with a fluorescence microscope (IX81, Olympus). Negative controls were performed by omitting the primary antibody.

**2 Results**

2.1 The pathological staining and IgA immunofluorescence staining

The kidney biopsy, wax specimen preparation, pathological staining and IgA immunofluorescence staining were performed by the Department of Pediatrics and Nephrology Pathology of the First Affiliated Hospital of Anhui Medical University, as shown in the supplementary materials (sFigure 1). The pathological staining including HE, PAS, PASM and Masson staining were performed in all IgAN puncture specimens, and the results were shown in sFigure 1A-D. With reference to the Lee's classification, the kidney pathology of 15 IgAN children was classified, including 1 case of grade Ⅰ, 6 cases of grade Ⅱ, 7 cases of grade Ⅲ, and 1 case of grade Ⅳ (sTable 2).

The immunofluorescence staining for IgA was performed on the kidney tissues of 15 children with IgAN. According to the fluorescence intensity grading, kidney IgA immunofluorescence staining were recorded from IgA (-) to IgA (++++). AS shown in sFigure 2A, there were 5 IgA (++) cases, 7 IgA (+++) cases and 3 IgA (++++) cases in this study. We analyzed the correlation between IgA deposition and Lee’s grade, as well as CCR2 expression. There was not the correlations between IgA deposition and Lee’s grade (r=0.2322, P=0.4050) (sFigure 2B), and CCR2 expression(r=0.2783, P=0.3152) (sFigure 2C).

2.2 Control immunohistochemistry staining

In order to verify the effectiveness of Polyclonal Rabbit anti-human CCR2 antibody (BioVision, Catalog: 3415R), the pre-immune rabbit serum replaced the primary antibody, as negative control (sFigure 3D), while rat kidney tissue was stained with BioVision CCR2 antibody as a positive control (sFigure 3C).

2.3 The relationship between CCR2 expression and clinical presentation

In this study, we analyzed the correlation between the relative OD value of CCR2 and the levels of 24h urine protein (Upro) and urinary RBC (URBC), and found no correlation between CCR2 expression and Upro (sFigure 4A, Spearman r=0.2686, P=0.3331) or URBC (sFigure 4B, Spearman r=0.2643, P=0.3412). Furthermore, there was no difference between low Lee’s grade group and high Lee’s grade group (*P*>0.05) (sFigure 4C and 4D).

2.4 The relationship between Oxford classification and inflammatory factors

According to Oxford classification, 15 IgAN children based on glomerular lesions were divided into 6 groups (MES classification), including M0E0S0, M0E0S1, M1E0S0, M1E0S1, M0-1E1S0 and M0-1E1S1. Lee’s grade and Oxford Classification scores characteristics of patients in the IgAN children were shown in sTable 2. The correlation between Oxford classification and inflammatory factors were analyzed, the correlation was only found between Oxford classification and CCR2 (r=0.6071, P=0.0164) (sFigure 5). As shown in sTable 3 and sTable 4, the relationship of Lee’s grade and Oxford classification were analyzed, and the correlation was found between Lee’s grade and Oxford Classification (MES) (r=0.5898; P=0.0162), or MET (r= 0.5709; P=0.0262) .

### Then on the basis of mesangial hyperplasia, 15 IgAN children were divided into 2 groups, (M0:≤0.5; and M1: > 0.5 according to Oxford classification). The correlation between mesangial hyperplasia and inflammatory factors were analyzed, the correlation was found between mesangial hyperplasia and CCR2 (r=0.7676, P=0.0008), or MCP-1(r=0.6979, P=0.0038), or TNF alpha(r=0.6298, P=0.0119) (sFigure 6).

### According to endothelial cell hyperplasia, 15 IgAN children were divided into 2 groups, (E0: No; and E1: Yes according to Oxford classification). The correlation between endothelial cell hyperplasia and inflammatory factors were analyzed, there was no correlation between endothelial cell hyperplasia and inflammatory factors (sFigure 7).

References

1. Wang X, Zhang K, Yang F, Ren Z, Xu M, Frank JA, Ke ZJ, Luo J (2018) Minocycline protects developing brain against ethanol-induced damage. Neuropharmacology 129:84-99.

2. Zou C, Suen PM, Zhang Y, Wang Z, Chan P, Leung PS, Zhang YA (2006) Isolation and in vitro characterization of pancreatic progenitor cells from the islets of diabetic monkey models. The international journal of biochemistry & cell biology 38:973-984.

**sTable 1** Demographics and clinical characteristics of patients in IgAN group and controls.

|  | IgAN (15)  Kidney biopsy | Control (8)  Kidney specimen | Control (12)  Blood and urine specimens | P value |
| --- | --- | --- | --- | --- |
| Sex(male/female) | 10/5 | 5/3 | 8/7 | 0.7836 |
| Age(years) | 11.67±1.76 | 10.75 ± 2.24 | 11.64 ± 3.17 | 0.8264 |
| Scr (μmol/L) | 77.38±8.25 |  | 61.25±6.56 | 0.0513 |
| URBC(μmol/L) | 689±427 |  | 6±10 | <0.001 |
| BUN (mmol/L) | 3.37±0.89 |  | 2.94±0.54 | 0.0827 |
| UPro (g/24h) | 2.08±0.39 |  | 0.06±0.02 | <0.001 |

**sTable 2** Lee’s grade and Oxford Classification scores characteristics of patients in the IgAN children.

|  | IgAN |
| --- | --- |
| Lee’s grade I- II | 7 |
| Lee’s grade III -V | 8 |
| M0/M1 | 4/11 |
| E0/E1 | 7/8 |
| S0/S1 | 14/1 |
| T0/T1/T2 | 15/0/0 |

**sTable 3** The relationship of Lee’s grade and Oxford classification.

| Lee’s grade | Oxford classification (MES) | | | | | | |
| --- | --- | --- | --- | --- | --- | --- | --- |
|  | M0E0S0 | M0E0S1 | M1E0S0 | M1E0S1 | M0-1E1S0 | M0-1E1S1 | Total |
| I | 1 | 0 | 0 | 0 | 0 | 0 | 1 |
| II | 3 | 0 | 1 | 0 | 2 | 0 | 6 |
| III | 0 | 0 | 2 | 1 | 4 | 0 | 7 |
| IV | 0 | 0 | 0 | 0 | 1 | 0 | 1 |
| V | 0 | 0 | 0 | 0 | 0 | 0 | 0 |
| Total | 4 | 0 | 3 | 1 | 7 | 0 | 15 |

Speaman r=0.5898; P=0.0162

**sTable 4** The relationship of Lee’s grade and Oxford classification.

| Lee’s grade | Oxford classification (MET) | | | | | |
| --- | --- | --- | --- | --- | --- | --- |
|  | M0E0T0 | M1E0T0 | M0-1E1T0 | M0-1E0T1-2 | M0-1E1T1-2 | Total |
| I | 1 | 0 | 0 | 0 | 0 | 1 |
| II | 3 | 1 | 2 | 0 | 0 | 6 |
| III | 0 | 3 | 4 | 0 | 0 | 7 |
| IV | 0 | 0 | 1 | 0 | 0 | 1 |
| V | 0 | 0 | 0 | 0 | 0 | 0 |
| Total | 4 | 0 | 3 | 1 | 7 | 15 |

Speaman r=0.5709; P=0.0262


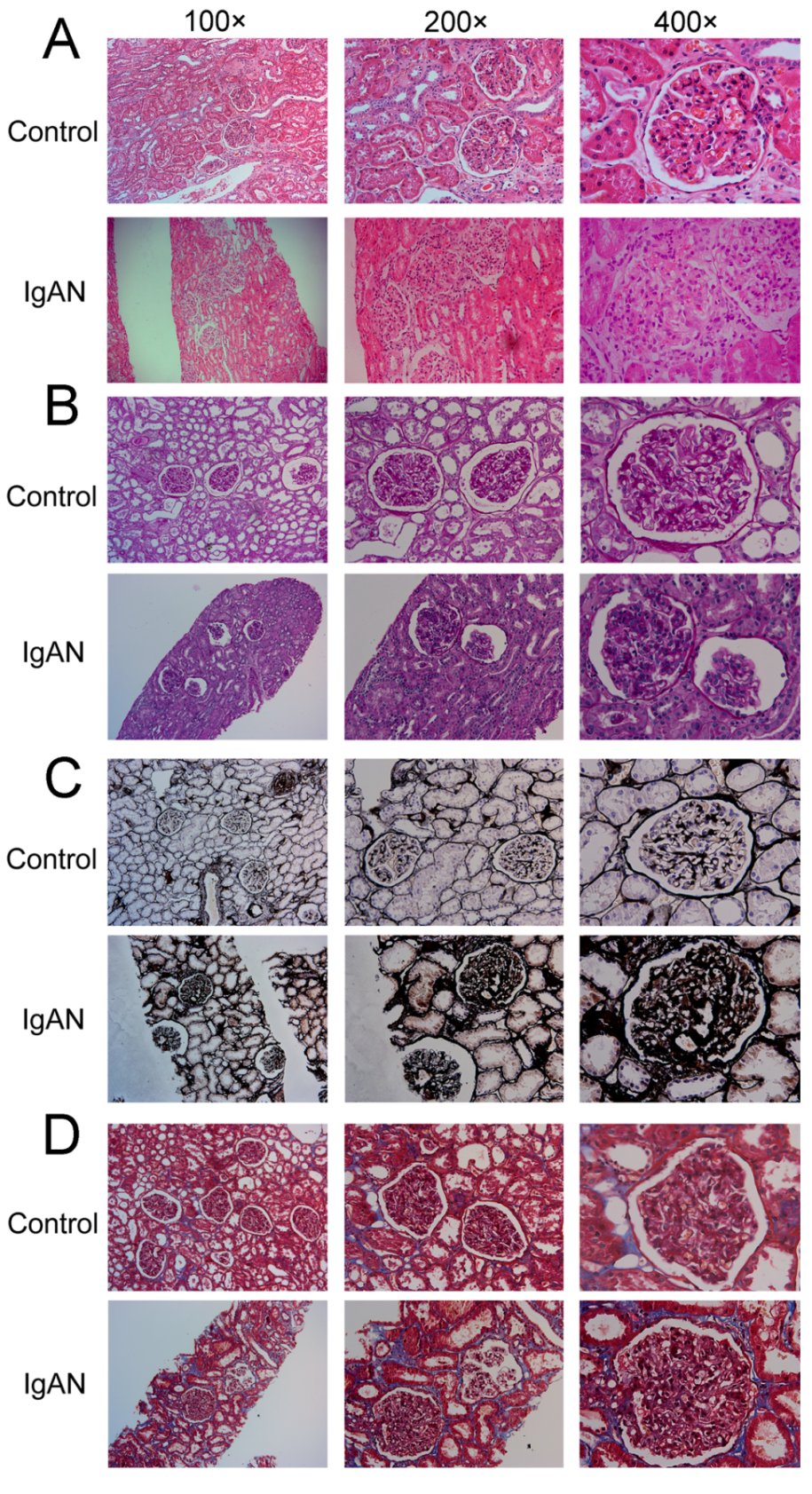


**sFigure 1** Kidney pathological staining in IgAN children

The pathological staining, including HE (**A**), PAS (**B**), PASM (**C**) and Masson staining (D) were detected in the kidney tissues of IgAN children and control group by immunohistochemistry. The Bar in left column of **A-D** was 200 µm, and in the middle and right column respectively was 100 µm and 50µm.


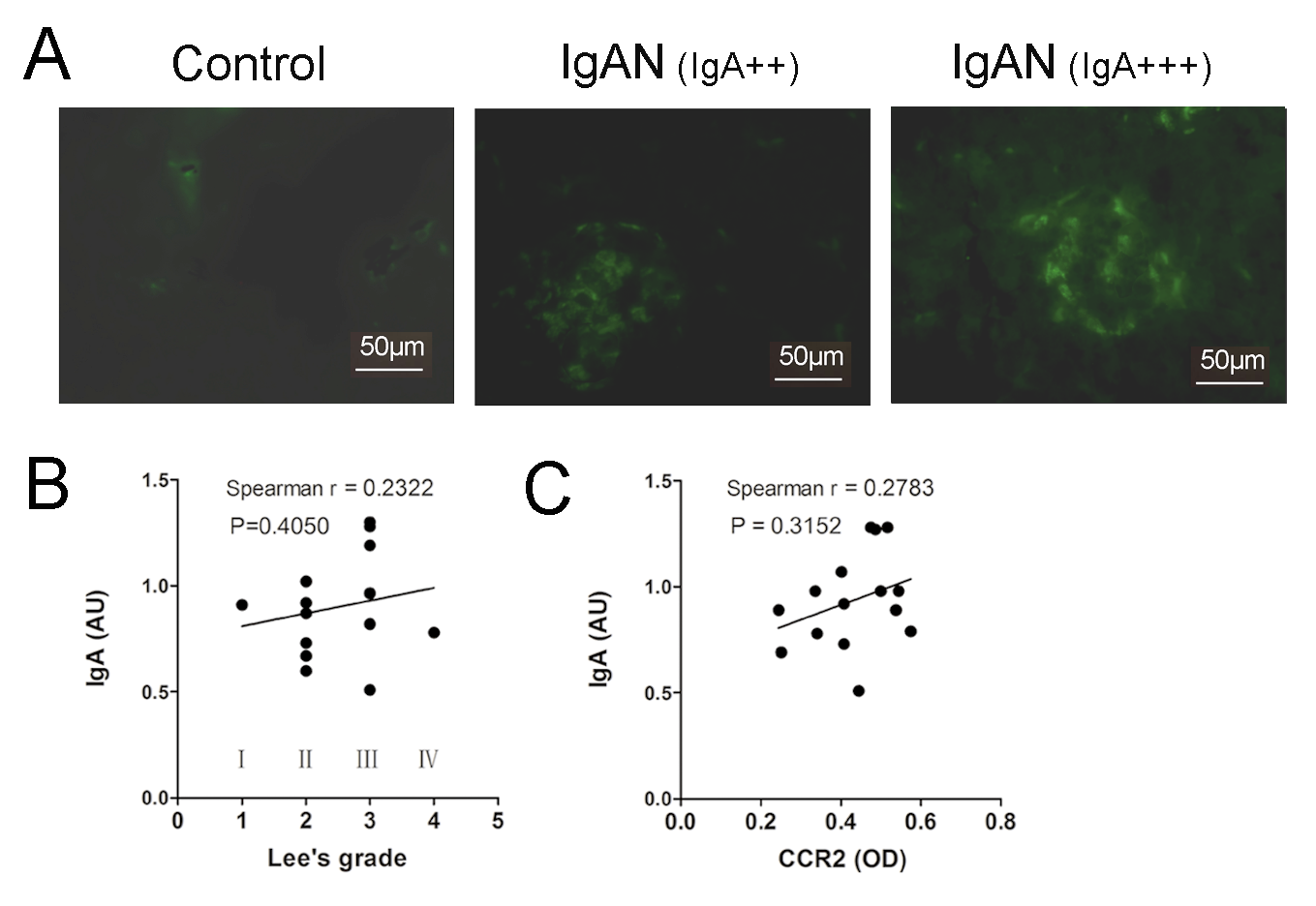


**sFigure 2** IgA immunofluorescence staining in IgAN children

IgA immunofluorescence staining was detected in the kidney tissues of IgAN children and control group by immunofluorescence (**A**). The Bar was 50 µm. The correlation between the average fluorescence intensity (AU) of IgA and Lee’s grade (**B**), or CCR2 expression (**C**), was analyzed.


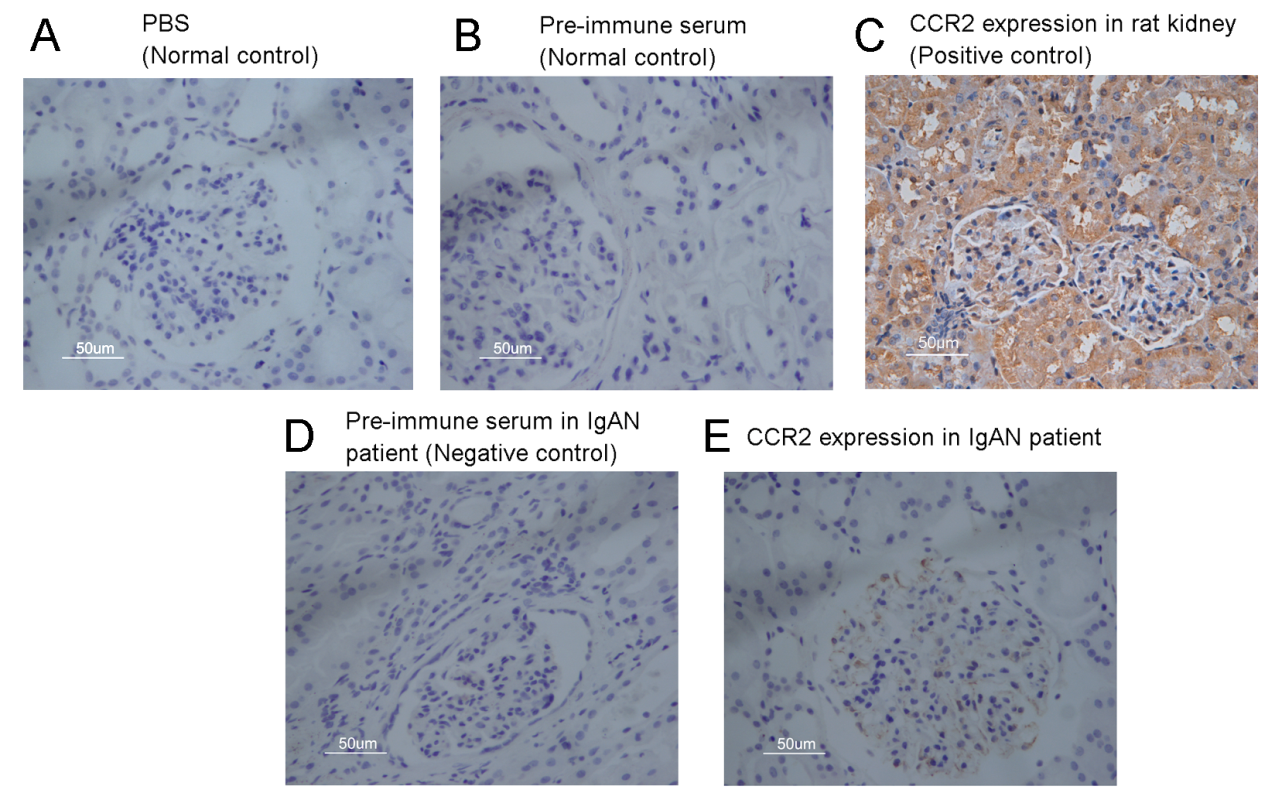


**sFigure 3** Control immunohistochemistry staining

Control staining was detected in control group (**A** and **B**), the kidney tissues of IgAN children (**D** and **E**) and rat kidney tissues (**C**) by immunohistochemistry. The Bar was 50 µm.


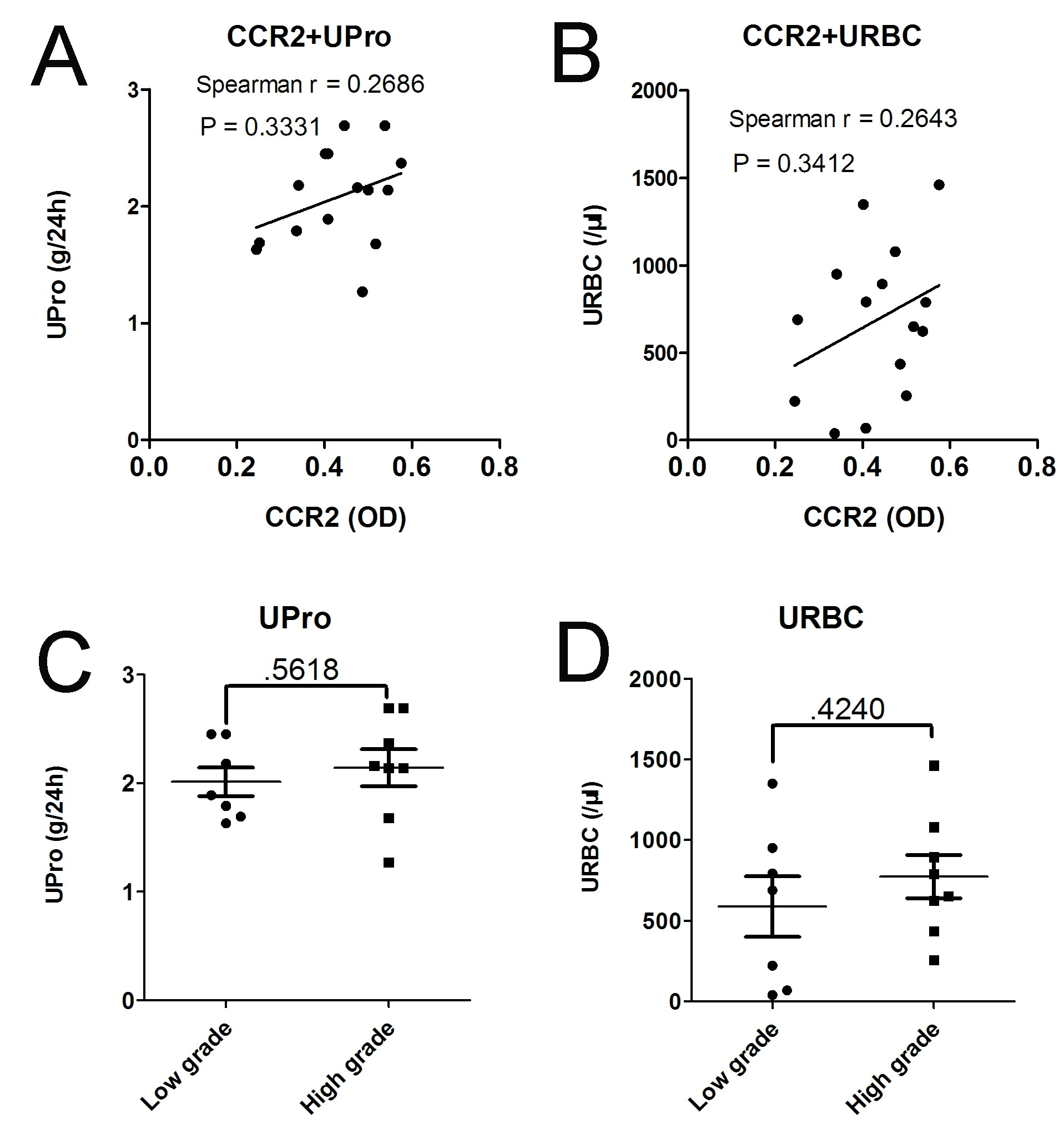


### sFigure 4 The relationship between CCR2 expression and clinical presentation

### The correlation between CCR2 exprssion and Upro (A), or URBC (B), and the difference between low grade and high grade group in Upro (C), or URBC (D), were analyzed.


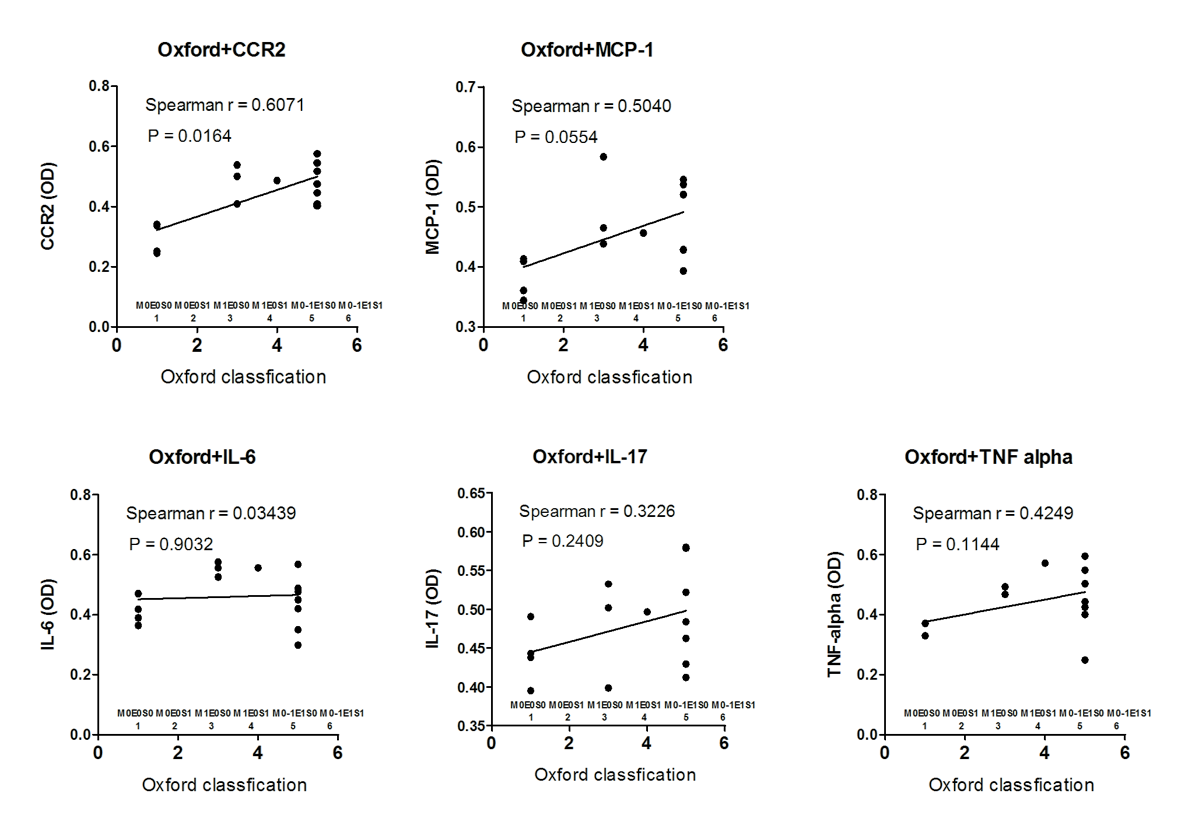


### sFigure 5 The correlation between Oxford classification and inflammatory factors

### The correlation between Oxford classification and inflammatory factors, including CCR2, MCP-1.IL-6, IL-17 and TNF alpha, were analyzed.

###
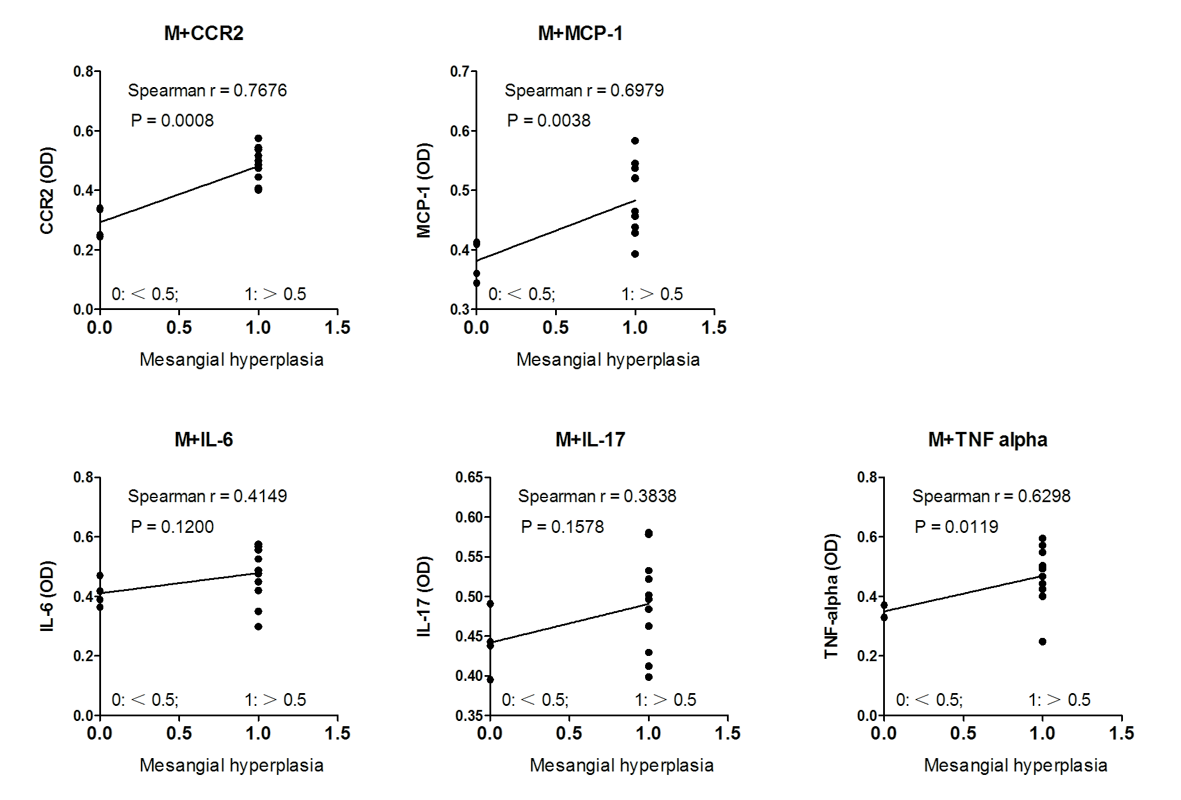


### sFigure 6 The correlation between mesangial hyperplasia and inflammatory factors

### The correlation between mesangial hyperplasia and inflammatory factors, including CCR2, MCP-1.IL-6, IL-17 and TNF alpha, were analyzed.

###
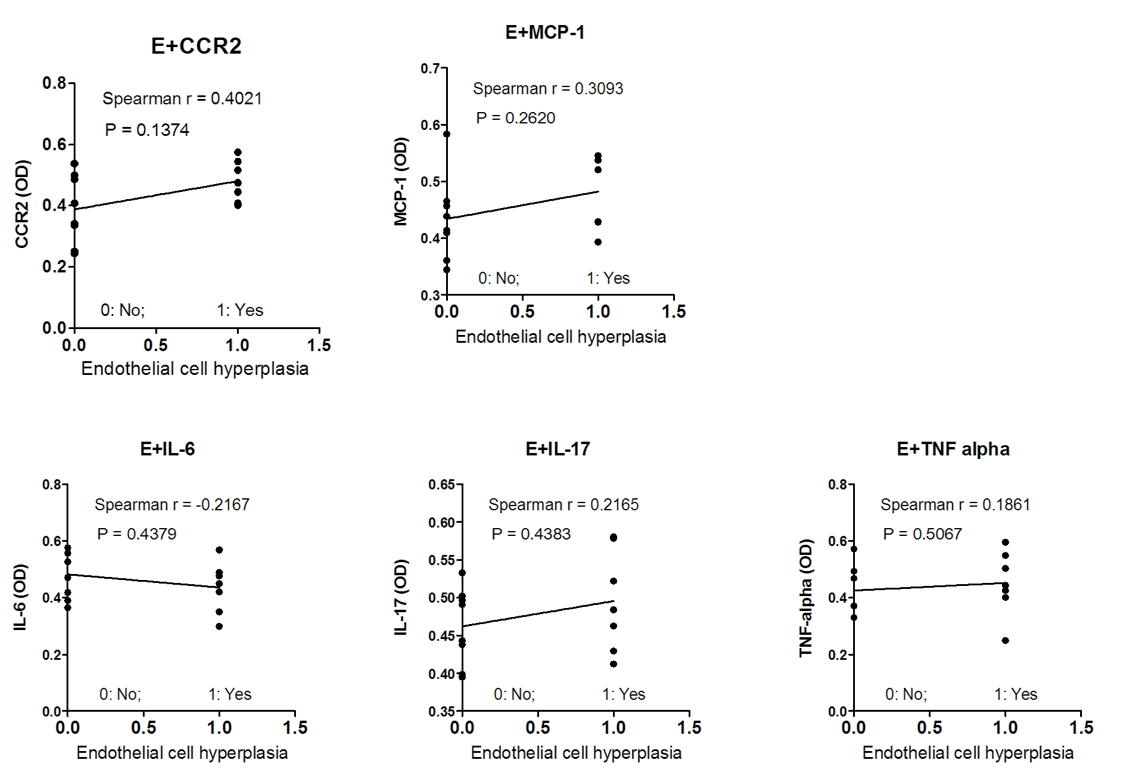
sFigure 7 The correlation between endothelial cell hyperplasia and inflammatory factors

### The correlation between endothelial cell hyperplasia and inflammatory factors, including CCR2, MCP-1.IL-6, IL-17 and TNF alpha, were analyzed.
